# The Book World of Medicine and Science

**Published:** 1895-01-26

**Authors:** 


### THE BOOK WORLD OF MEDICINE AND SCIENCE.

A TEXT BOOK OF PATHOLOGY, SYSTEMATIC AND PRACTICAL. Vol. 2. Parts I. and II. By D. J. HAMILTON, M.B., F.R.S., Edin., Professor of Pathology, Aberdeen University. (London: Macmillan and Co. 1894.)

We welcome the issue of this excellent text book, although so long a time has elapsed since the appearance of the first volume.

Part I. continues the consideration of the pathology of the special tissues and organs, viz , the nares, larynx and trachea, pleura and lung, liver, kidney and bladder, urethra, male and female organs of generation, lips, mouth, tongue and pharynx, œsophagus and stomach. Under the head of "Pleura" we should like to have heard more of the pathological changes seen in empyema discussed, especially as they differ in the child and adult; while on the other hand under "Lungs," and in some other places (Part II.), the physiology of the normal respiratory sounds, the physiology of the other organs, especially those of the nervous system, are discussed at too great a length even for the elucidation of the pathological changes. In this manner we think there is much speculative matter introduced, to the exclusion of so many important details of pathological anatomy which we would rather have seen in a work devoted to pathology. Nevertheless, such instinct gives much food for reflection, and renders the work a very suggestive one. The term "Tuberculous" might with advantage be substituted for "Tubercular," as is usually done at the present day. While the diseases of the bladder, ureter, and kidneys are carefully treated, we fail, under the head of "Testis," to find any mention of tuberculosis of the tunica vaginalis, which is probably a more common condition in young life than is generally supposed.

Part II. contains the diseases of the intestine, nervous system, thyroid gland, supra-renal capsules, spleen, bones, joints, skin, &c. In the section on the nervous system, Duret's table of the areas of distribution of the encaphalic blood-vessels are serviceable, and the cranial topography and the structure of the brain are exceedingly well treated. With regard to the latter, it is well known that Professor Hamilton was one of the first to make sections of the whole cerebrum, and these sections, here reproduced, are of peculiar value to the pathologist as well as to the student. For by this method only can the complex course of the different nerve fibres be accurately studied, and changes in the nervous system cannot well be left unpassed. The pathology of chorea is dismissed in a few lines, saying that all theories of this disease have been weighed and found wanting. The sections on the mamma and skin give a short but succinct account of the diseases of these organs. This volume concludes with two chapters concerning malformation, systematic bacteriology, and on animal parasites met with in man, and with a chapter on animal heat and fever. To the bacteriological section too much praise cannot be given. The diagrams of microscopical sections, as in the first volume, faithfully represent the great and arduous work of the eminently practical teacher. Those representing the lesions of the nervous system deserve special attention. It is only to be regretted that the diagrams of the macroscopical aspects of disease are so crude. They are unworthy of the rest of this otherwise beautifully illustrated work. The plan of indicating by numbers in the text the name of the publication, and inserting a key at the end of the book, is not a particularly happy one, and does not, as far as we can see, avoid much useless repetition. The remarks which we notice upon treatment, however accurate and even useful, are out of place in a book upon scientific pathology.

In spite of the minor defects noted, we have nothing but unqualified praise for this work, which will doubtless maintain the high position attained by the first volume as a most valuable book to teachers, students and practitioners. It ranks as one of the most estimable of the kind hitherto published.

